# Morphological and molecular genetic analysis of the genus Iris L. polymorphism in the Republic of Bashkortostan and the Orenburg Region

**DOI:** 10.18699/vjgb-26-16

**Published:** 2026-03

**Authors:** E.A. Aukhadieva, A.G. Blinov, E.I. Vshivtseva, Z.Kh. Shigapov, A.R. Ishbirdin, Ya.M. Golovanov, A.V. Kryukova

**Affiliations:** Ufa Research Institute of Occupational Health and Human Ecology, Ufa, Russia; Institute of Cytology and Genetics of the Siberian Branch of the Russian Academy of Sciences, Novosibirsk, Russia; Institute of Cytology and Genetics of the Siberian Branch of the Russian Academy of Sciences, Novosibirsk, Russia Novosibirsk State University, Novosibirsk, Russia; South-Ural Botanical Garden-Institute – Subdivision of the Ufa Federal Research Centre of the Russian Academy of Sciences, Ufa, Russia; Ufa University of Science and Technology, Ufa, Russia; South-Ural Botanical Garden-Institute – Subdivision of the Ufa Federal Research Centre of the Russian Academy of Sciences, Ufa, Russia; South-Ural Botanical Garden-Institute – Subdivision of the Ufa Federal Research Centre of the Russian Academy of Sciences, Ufa, Russia

**Keywords:** Iris, taxonomy, taxonomic indicators, nucleotide sequences, trnL-trnF chloroplast DNA sequence, phylogenetic tree, Iris, таксономия, таксономические индикаторы, нуклеотидные последовательности, trnL-trnF последовательность хлоропластной ДНК, филогенетическое древо

## Abstract

Iris is a cosmopolitan genus comprising 200 to 340 species distributed throughout the Northern Hemisphere. Although Iris is the most diverse group in the family Iridaceae, there are many uncertainties regarding its taxonomic composition and systematics. The aim of this study was to search for taxonomically significant morphological characters of the generative and vegetative spheres and molecular markers with subsequent assessment of their informativeness in identifying phylogenetic relationships and compliance with the most relevant modern classification systems of the genus Iris. As a result of constructing the structure of variability of morphometric parameters of 11 species, 10 taxonomic indicators were identified that were common to the analyzed taxa and were characterized by relatively low total and coordinated variability: length and width of the outer perianth lobes, length and width of the inner perianth lobes, length of the filament, anther and pistil, fruit width, as well as seed length and width. Nucleotide sequences of trnL-trnF fragments of chloroplast DNA were established for 13 samples of four species of wild flora of the Republic of Bashkortostan and the Orenburg Region: Iris pumila L., I. scariosa Willd. ex Link., I. pseudacorus L., I. sibirica L. The obtained sequences were used to construct a phylogenetic tree together with trnL-trnF sequences of seven more iris species extracted from the database. The tree contained five clusters: (1) I. pumila, I. scariosa; (2) I. pseudacorus, I. setosa Pall. ex Link; (3) I. lactea Pall.; (4) I. sibirica, I. sanguinea Hornem.; (5) I. spuria L., I. xanthospuria Mathew & Baytop., I. foetidissima L., I. sintenisii Janka. By the composition of their species, the identified clusters almost completely coincided with the clusters found during the morphological analysis. To confirm the obtained results, a phylogenetic analysis of the species of interest was performed on two more chloroplast sequences available in the database: matK and trnS-trnG. Clustering of the studied species on trnS-trnG and matK completely coincided with clustering on trnL-trnF. Thus, we can state that the morphological features identified for the Iris generic complex work in the taxonomic direction. The analysis also showed that I. scariosa from natural populations of the Republic of Bashkortostan and the Orenburg Region were identified correctly.

## Abstract (ru)

Iris L. (Iridaceae Juss.) – это космополитный род, включающий от 200 до 340 видов, распространенных по всему Северному полушарию. Хотя Iris является самой разнообразной группой в семействе Iridaceae, существует множество неопределенностей относительно его таксономического состава и систематики. Цель настоящего исследования – поиск молекулярных маркеров и таксономически значимых морфологических признаков генеративной и вегетативной сферы и оценка их информативности для выявления филогенетического родства представителей рода Iris. Образцы ирисов взяты из природных популяций Республики Башкортостан и Оренбургской области, а также получены из ботанических садов Санкт-Петербурга, Бонна, Байройта и Брно. В результате построения структуры изменчивости морфометрических показателей 11 видов найдено 10 таксономических индикаторов, общих для анализируемых таксонов и характеризующихся относительно низкими общей и согласованной изменчивостью: длина и ширина наружных долей околоцветника, длина и ширина внутренних долей околоцветника, длина тычиночной нити, пыльника и пестика, ширина плода, а также длина и ширина семени. Установлены нуклеотидные последовательности trnL-trnF фрагментов хлоропластной ДНК для 13 образцов четырех видов дикорастущей флоры Республики Башкортостан и Оренбургской области: Iris pumila L., I. scariosa Willd. ex Link., I. pseudacorus L., I. sibirica L. Полученные последовательности были использованы для построения филогенетического дерева совместно с trnL-trnF последовательностями еще семи видов ирисов, извлеченных из базы данных. На филогенетическом древе формируется пять групп (кластеров): 1) I. pumila L., I. scariosa Willd. ex Link; 2) I. pseudacorus L., I. setosa Pall. ex Link; 3) I. lactea Pall.; 4) I. sibirica L., I. sanguinea Hornem.; 5) I. spuria L., I. xanthospuria Mathew & Baytop., I. foetidissima L., I. sintenisii Janka. Выявленные кластеры по составу входящих в них видов практически полностью совпадают с кластерами, обнаруженными при морфологическом анализе. Для подтверждения полученных результатов проведен филогенетический анализ интересующих видов еще на двух хлоропластных последовательностях, доступных в базе данных: matK и trnS-trnG. Кластеризация исследованных видов на trnS-trnG и matK полностью совпадает с кластеризацией на trnL-trnF. Таким образом, можно констатировать, что выявленные для родового комплекса Iris морфологические признаки демонстрируют значимую диагностическую ценность при проведении таксономических исследований.

## Introduction

The genus Iris L. is the largest and most cosmopolitan in the family Iridaceae, distributed mainly in the temperate zones of the Northern Hemisphere, and includes from 200 to 340 species (Dorofeeva, Zhurbenko, 2020). Despite significant progress in the study of the genus Iris, there are still many uncertainties regarding its taxonomic composition and systematics. The genera boundaries of irises are controversial, and recent data appear to favor much narrower boundaries (Crespo et al., 2015; Boltenkov et al., 2020). In addition, its composition is periodically supplemented with new described species (Zhao, 1992; Mitic, 2002; Alexeeva, 2013a), which is often associated with morphological variability and, as a consequence, repeated descriptions of species with a wide range (Boltenkov et al., 2018a).

Classifications of the genus Iris are most often based on anatomical, morphological and cytogenetic characteristics (Doronkin, Krasnikov, 1984; Mathew, 1989; Makarevich et al., 2001), as well as on the results of molecular biological and biochemical studies (Dorogina et al., 2012; Weber et al., 2020). The difficulty is that systematics have both, broad and narrow understandings of the genus Iris. There are no commonly accepted classification, and the most popular classification schemes (Rodionenko, 1961; Mathew, 1989; Doronkin, 2006) have differences not only in position of individual species, but also higher taxonomical units – subgenera and sections. Attempts at resolving those contradictions using modern methods often provide ambiguous results. Molecular RAPDanalysis by I.F. Makarevich and colleagues (2001) showed greater agreement with the system of G.I. Rodionenko (1961) in establishing phylogenetic relationships

Studies of Siberian species have revealed unexpected groupings: species from Xyridion and I. sibirica (Limniris) formed one group, while I. lactea and I. setosa (Limniris) formed another group with species from subgenus Iris. These data are contradicting with the existing schemes, especially in regard to polymorphic subgenus Limniris. The lack of consensus on the composition of the genus Iris among modern researchers requires further comprehensive studies to clarify phylogenetic relationships of the species included in this genus.

There are publications in the literature on how assessment of the structure of morphological variability can be used in biology for classifying morphological characteristics of some plant and animal species according to the ratio of general and coordinated variability. The authors identify four groups of indicators: ecological-biological systemic, biological, genetic (taxonomic), and ecological indicators (Rostova, 2002; Ishbirdin et al., 2005).

Ecological and biological systemic indicators are characterized by high general and coordinated variability, strong dependence on environmental conditions, and the ability to induce coordinated changes in the entire morphosystem of an organism (number of buds, shoot length in C. rubra, leaf blade length in Triticum aestivum L.).Biological indicators have moderate environmental dependence, low total and high coordinated variability. They also determine the morphostructure of the plant (e. g., shoot height, leaf parameters in Cephalanthera rubra, the number of metameres in the vegetative part of the annual shoot in Helianthus annuus).

Genetic (taxonomic) indicators are characterized by low general and coordinated variability, high autonomy and weak dependence on external conditions (for example, the number of leaves in C. rubra), and are the most informative for taxonomic studies

Ecological indicators exhibit strong, relatively independent variation, and their changes are only mildly correlated with the overall system of the organism, but they are sensitive even to minor external influences (for example, the number of immature flowers, signs of branching in Rhinanthus L.). For species such as H. annuus L., C. rubra L., Panicum miliaceum L., and a number of other flowering plants, these traits have already been identified. For the Iris genus complex, a correlation analysis of morphological traits to identify these indicators was conducted for the first time.

DNA polymorphisms in noncoding regions of chloroplast genome are successfully used to establish phylogenetic relationships between species of the genus Iris (Pleines et al., 2009). Among the most often used regions of chloroplast DNA are the trnL intron and the trnL-trnF intergenic spacer, as well as other variable regions of chloroplast DNA including: atpBrbcL, trnS-trnG, and trnH-psbA intergenic spacers (Kozyrenko et al., 2009; Boltenkov et al., 2016).

The aim of the current study is to search for taxonomically significant morphological features of the generative and vegetative spheres and molecular markers, followed by an assessment of their informativeness in identifying phylogenetic relationships and compliance with the most relevant modern classification systems of the genus Iris.

## Materials and methods

Plant material. The objects of morphological studies were wild-growing representatives of species of the genus Iris (I. pseudacorus L., I. sibirica L., I. pumila L., I. scariosa Willd. ex Link), collected from natural populations of Bashkortostan Republic and Orenburg Region, and introduced to the collection area of the South-Ural Botanical Garden-Institute of the Ufa Federal Research Center of the Russian Academy of Sciences in 2019–2021. As well as from seed material grown in obtained from delectuses from the botanical gardens of St. Petersburg (I. lactea Pall., I. setosa Pall., I. halophilа Pall.), Bonn (I. sanguinea Hornem., I. spuria L.), Bayreuth (I. сarthaliniae Fomin) and Brno (I. graminea L.). Details on species used, collection locations and geographical coordinates are listed in Table 1.

**Table 1.**
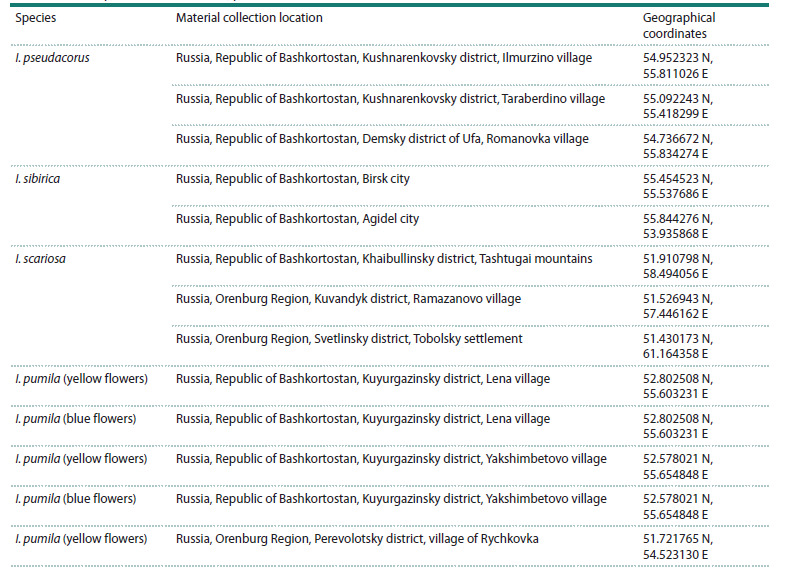
Iris samples used in this work, places and time of their collection

Due to the identification of a large number of controversial issues regarding the taxonomy of species of the genus Iris L., in this work we focused on the classifications of three authors: B. Mathew (1989), G.I. Rodionenko (1961, 2013), and V.M. Doronkin (2006).

Morphological analysis. Morphometric parameters were recorded for 25 plants of each species. To assess variability, the following parameters were analyzed: generative shoot height, leaf length and width, perianth segment length and width, reproductive element length (pistil and stamen) during the flowering phase, and fruit and seed length and width after full ripening. Measurements of morphometric parameters of shoots, flowers and fruits were carried out using a ruler, and those of seeds, using a Levenhuk DTX 90 microscope (Golubev, 1962). Standard statistical processing of the obtained data was carried out using the programs MS Excel and IBM SPSS Statistics 21 (Zaitsev, 1984; Dospekhov, 1985). The arithmetic mean, standard error of the mean, and standard deviation were calculated. To assess intrapopulation variability of morphological traits, the coefficient of variation (СV, %) was calculated (Zaitsev, 1990).

A comparison of biometric indicators was conducted to determine the statistical significance of their differences across 2019–2021 years. The values of the studied indicators were tested for normal distribution using the Kolmogorov– Smirnov test. To compare independent samples with a normal distribution, a one-way analysis of variance was used, and for samples that do not obey the law of normal distribution, the Kruskal–Wallis test was used. The analysis showed that for all the parameters studied, the difference is insignificant at a significance level of W = 5 %, which makes it possible to assess the structure of variability of morphological characteristics and classify them into groups

The structure of trait variability is constructed using the method of N.S. Rostova (2002), using the programs MS Excel and IBM SPSS Statistics 21. Morphological traits are coordinated in the space of total (the trait’s coefficient of variation) and consistent (the squared correlation coefficient averaged over the trait) variability. According to their indicator role, traits are divided into three groups: taxonomic (genetic), biological, and ecological-biological systemic.

Isolation of total DNA, PCR amplification and determination of nucleotide sequences and phylogenetic analysis. Total DNA was isolated using the DNeasy Plant Mini Kit (QIAGEN, Germany) according to the manufacturer’s protocol. 50–100 mg of dried leaves from each plant were used for DNA extraction. The quantity and quality of the isolated DNA were determined using a NanoDrop2000 spectrophotometer (Thermo Scientific, USA) and electrophoretic separation in a 1 % agarose gel containing ethidium bromide (0.5 mg/ml) in 1xTAE.

PCR amplification was performed as described in (Makarevich et al., 2003) with primers specific for the trnL/trnF chloroplast genes:

trnL (5′-CGAAATCGGTAGACGCTACG-3′); trnF (5′-ATTTGAACTGGTGACACGAG-3′).

The reaction was performed in a 20 μl volume using Bio- Master LR HS-PCR-Color (Biolabmix, Novosibirsk, Russia) with 10 pmol of each primer and 30 ng of total DNA. The resulting PCR fragments were separated on a 1 % agarose gel and isolated from the gel using the QIAquick Gel Extraction Kit (QIAGEN, Germany), followed by sequencing. Sequencing reactions were performed using 200 ng of DNA fragment and the BigDye Terminator v3.1 Cycle Sequencing Kit (Thermo Scientific, USA) on an ABI 3130XL genetic analyzer (Applied Biosystems, USA) at the Genomics Center of the Siberian Branch of the Russian Academy of Sciences (http://www. niboch.nsc.ru/doku.php/corefacility). The nucleotide sequences of the trnL-trnF fragments of chloroplast DNA are presented in GenBank (No. PV335670–PV335682).

For phylogenetic analysis, trnL-trnF iris chloroplast DNA sequences retrieved from the GenBank database and obtained in this study were used. Nucleotide sequence alignment was performed using MAFFT v7.312 (Katoh, Standley, 2013). Suitable nucleotide substitution model was selected using the Bayesian Information Criterion. The phylogenetic tree was constructed using the maximum likelihood method with the Jukes–Cantor model in the IQ-tree program (Trifinopoulos et al., 2016). The reliability of the constructed tree was tested using the Bootstrap method with a number of repetitions equal to 1,000.

## Results

**Analysis of morphometric parameters**

Morphological data for three years of research were analyzed. Biometric indicators were compared for statistical significance of differences between the years. The analysis showed that for all studied parameters, the differences were insignificant at a significance level of W = 5 %. This allowed us to assess the structure of morphological trait variability based on the ratio of total and consistent variability and classify them into groups. Generalized morphometric parameters of studied species are listed in the Table 2.

**Table 2.**
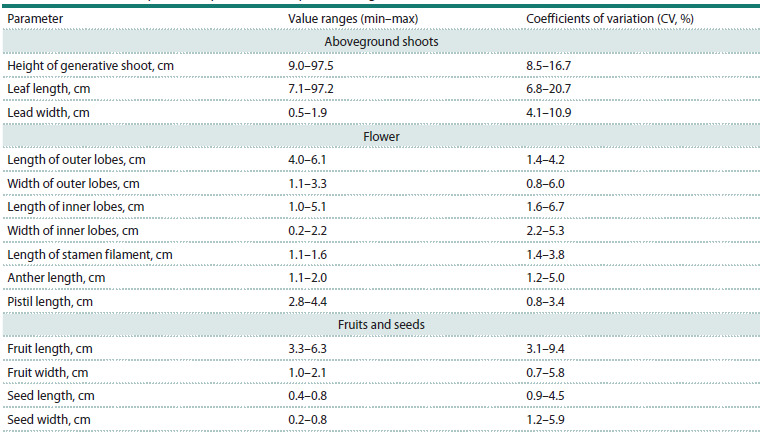
Generalized morphometric parameters of species from genus Iris (2019–2021)

An analysis of the variability of morphometric parameters of species of the genus Iris revealed the following patterns:

1. The highest variability level is typical for the length of a leaf blade (CV reaches 20.7 %) and the height of generative shoots (CV up to 16.7 %). 2. The most stable indicators – width of the inner perianth lobes (CV = 2.2–5.3 %) and pistil length (CV = 0.8–3.4 %), demonstrating the smallest spread of the variation coefficient values. 3. Fruit and seed parameters show higher variability in fruit length (CV up to 9.4 %), compared to its width (CV up to 5.8 %), and moderate variability in seed size (CV in the range of 0.9–5.9 %).

**Fig. 1.**
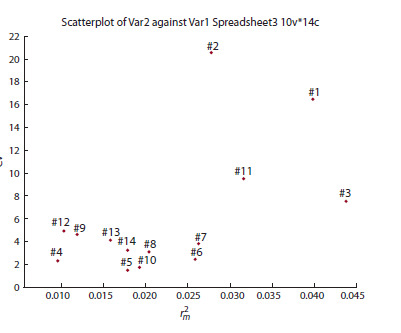
Structure of variability of morphological characteristics of I. pseudacorus. The X-axis shows the consistent variability, the Y-axis shows the total variability. 1 – height of generative shoot, 2 – leaf length, 3 – leaf width, 4 – length of outer perianth lobes, 5 – width of outer perianth lobes, 6 – length of inner perianth lobes, 7 – width of inner perianth lobes, 8 – length of filament, 9 – length of anther, 10 – length of pistil, 11 – length of fruit, 12 – width of fruit, 13 – length of seed, 14 – width of seed.

Morphological features are coordinated in the space of general (coefficient of variation of the feature) and consistent (squared correlation coefficient averaged for the feature) variability. Graphs were compiled showing the structure of morphological trait variability. As an example, Figure 1 shows the structure of variability for I. pseudacorus. Variability structure for I. sibirica, I. sanguinea, I. setosa, I. halophila, I. graminea, I. сarthaliniae, I. spuria, I. pumila, I. scariosa, I. lactea is given in the Supplementary Materials1

Supplementary Materials are available in the online version of the paper: https://vavilovj-icg.ru/download/pict-2026-30/appx10.pdf


In the current study, according to their indicator role, the features were divided into three groups: taxonomic (genetic), biological and ecological-biological (Fig. 1). As a result of constructing the variability structure, 10 taxonomic indicators were identified that are common to the analyzed taxa and characterized by relatively low total (CV = 0.8–6.0 %) and consistent ( r m 2 = 0.005–0.078) variability: the length and width of the outer perianth lobes, the length and width of the inner perianth lobes, the length of the filament, anther and pistil, the width of the fruit, and the length and width of the seed.

**Fig. 2.**
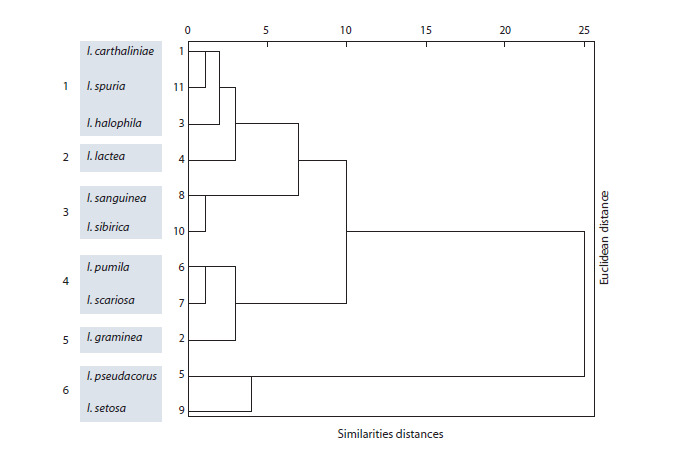
Dendrogram of differences and similarities of species of the genus Iris by taxonomic indicators.

To establish the relationship of the studied representatives of the genus Iris, a cluster analysis (hierarchical classification, Ward’s method) was carried out and a dendrogram of the differences and similarities between the species was constructed based on the identified diagnostic markers (Fig. 2). The species in the dendrogram can be grouped into six clusters:

1. I. carthaliniae, I. halophila, I. spuria, representatives of subgenus Xyridion (Rodionenko, 1961) or Limniris (Mathew, 1989). 2. I. lactea, subgenus Limniris (Rodionenko, 1961; Mathew, 1989) or Eremiris (Doronkin, 2006). 3. I. sibirica, I. sanguinea, subgenus Limniris. 4. I. pumila, I. scariosa, subgenus Iris. 5. I. graminea, subgenus Xyridion or Limniris. 6. I. pseudacorus, I. setosa, subgenus Limniris.

**Molecular phylogenetic analysis of species of the genus Iris**

**Fig. 3.**
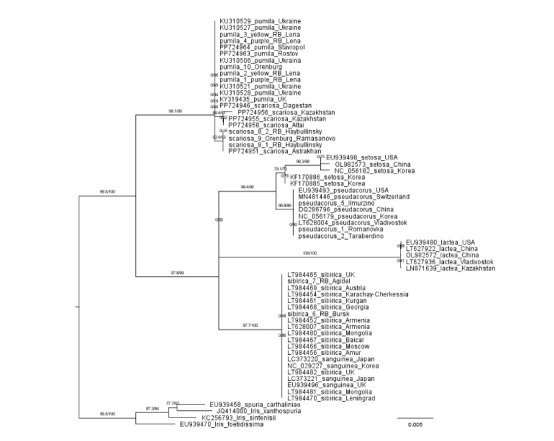
Phylogenetic tree constructed from iris trnL-trnF cpDNA sequences using the ML method. Collection locations and nucleotide sequence numbers from Genbank database are marked on the tree.

During the current project, the nucleotide sequences of trnLtrnF fragments of chloroplast DNA were established for 13 samples of 4 species of irises: I. pumila, I. pseudacorus, I. sibirica, I. scariosa (Table 1). The obtained sequences were used to construct a phylogenetic tree together with the trnLtrnF sequences of 7 more iris species retrieved from the database: I. setosa, I. lactea, I. sanguinea, I. spuria, I. xanthospuria Mathew & Baytop., I. foetidissima L., I. sintenisii Janka. As a result of this study, trnL-trnF sequences from 63 accessions of 11 species were analyzed. The resulting phylogenetic tree is presented in Figure 3. The collection locations of the studied iris accessions are marked on the tree.

There are five clusters on the tree: 1. I. spuria, I. xanthospuria, I. sintenisii, I. foetidissima. 2. I. lactea. 3. I. sibirica and I. sanguinea. 4. I. pumila and I. scariosa. 5. I. pseudacorus and I. setosa

These clusters, in terms of the composition of their species, almost completely coincide with the clusters found during morphological analysis (Fig. 2).

**Fig. 4.**
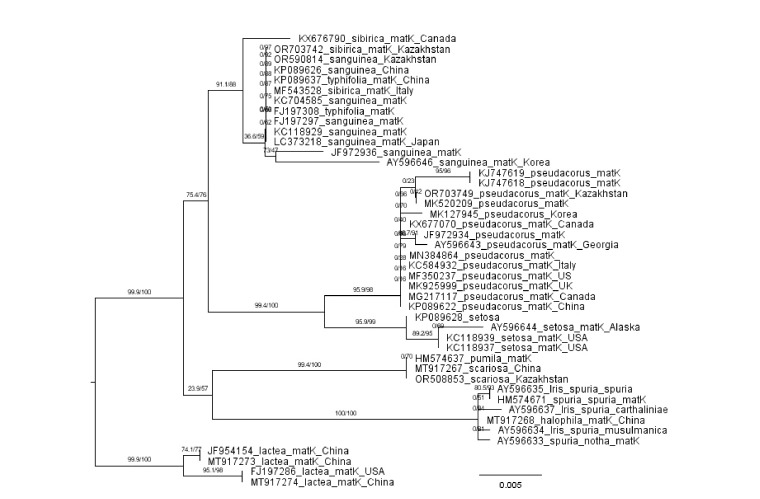
Phylogenetic tree constructed from the matK nucleotide DNA sequences of irises using the ML method Collection locations and nucleotide sequence numbers are marked on the tree.

To confirm our results, we conducted a phylogenetic analysis of the Iris species of interest using two additional chloroplast sequences available in the NCBI database: matK and trnStrnG. The dendrogram, obtained using matK sequences, showed that the samples split in five groups, similar to the grouping for trnL-trnF sequences (Fig. 4):

1. I. spuria and I. halophila. 2. I. lactea. 3. I. sibirica and I. sanguinea. 4. I. pumila and I. scariosa. 5. I. pseudacorus and I. setosaHowever, there are minor differences between the trnLtrnF and matK trees. In the pairs I. pumila/I. scariosa, I. sibirica/ I. sanguinea, and I. halophila/I. spuria, the matK sequences do not confirm species differences. While the species I. pseudacorus and I. setosa are reliably different from each other. If interpopulation differences in the trnL-trnF sequences were found in only two species (I. scariosa and I. setosa), such differences in the matK sequences are present in a larger number of species: I. setosa, I. pseudacorus, I. sibirica, I. sanguinea, and I. lactea. They may also be present in I. pumila and I. scariosa, but we found only three matK gene sequences from these species in the database.

**Fig. 5.**
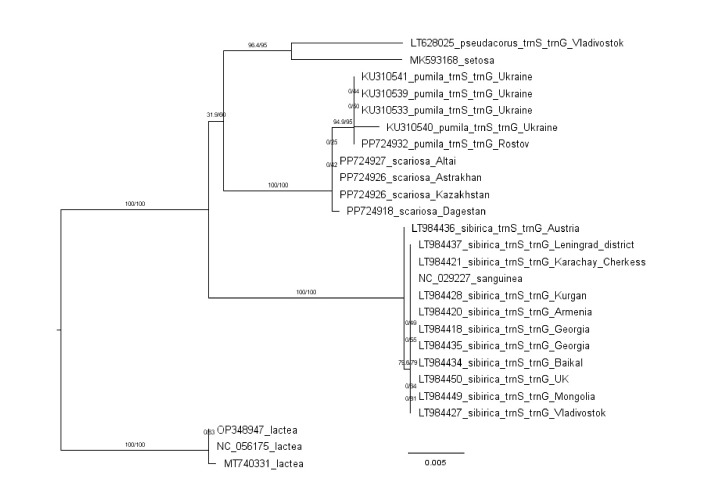
Phylogenetic tree constructed from Iris trnS-trnG cpDNA sequences using the ML method. Collection locations and nucleotide sequence numbers are marked on the tree.

There is less information about the trnS-trnG sequences in the database than about the previous two. Figure 5 shows a tree constructed from the trnS-trnG sequences. It contains four out of the five clusters obtained in the previous trees: (1) I. lactea; (2) I. sibirica and I. sanguinea; (3) I. pumila and I. scariosa; (4) I. pseudacorus and I. setosa. Cluster five is missing because there are no trnS-trnG sequences of I. spuria and I. halophile in the database. We will not go into detail on the analysis of the trnS-trnG tree due to the small number of samples for some species. However, the main conclusion is clear: the clustering of the studied species on the trnS-trnG tree is completely consistent with that on the trnL-trnF and matK trees.

## Discussion

**Morphometric parameters**

A comparative analysis of the morphometric parameters of the studied species with published data (Volkova, 2010) revealed a high degree of correspondence in the aboveground traits for most species, with minor deviations observed in some taxa. The detected differences are presumably determined by the specific cultivation conditions, which are supported, in particular, by the influence of temperature amplitude on the variability of morphometric parameters of the generative shoot and leaf in I. pumila from populations of the Lower Volga region and the Southern Urals (Kashin et al., 2022).

Based on the structure and morphometry of the perianth, the species were divided into conventional groups: (1) with long narrow segments (I. carthaliniae, I. halophila, I. spuria, I. graminea, I. lactea); (2) with large outer and reduced inner segments (I. pseudacorus, I. setosa); (3) with large broad segments (I. sibirica, I. sanguinea); (4) with medium-sized segments (I. pumila, I. scariosa), which indicates a high degree of reliability and validity of modern classifications of the genus.

A comparative analysis of the morphometric characteristics of fruits and seeds of Iris from natural populations of the Republic of Bashkortostan and the Orenburg Region with published data from other regions (Alexeeva, 2020) revealed moderate geographic polymorphism. In terms of seed size, the smallest seeds are characteristic of populations of I. sibirica (Saratov Region), I. halophila and I. pumila (Volgograd Region); a large-seeded form was recorded in I. pseudacorus (Primorsky Krai). Regarding fruit parameters, a slight reduction was observed in I. setosa, I. graminea, I. spuria, and I. pumila (Belgorod Region). The detected slight variability in size, as well as in the pigmentation of reproductive structures, reflects micropopulation differentiation of the species and local adaptive responses to growing conditions, ensuring their successful reproduction under diverse environmental conditions.

The coefficient of variation of morphological traits serves as a useful tool for the preliminary assessment of the stability of morphological characteristics, however, it is incorrect to judge the taxonomic significance of traits solely on the basis of the coefficient of variation. Taxonomic decisions must be based on a synthesis of morphological, genetic, and ecological data. For the genus Iris, this is particularly relevant due to its polymorphism and tendency toward hybridization. The method for identifying taxonomic characters proposed by N.S. Rostova (2002) is based on the analysis of correlation pleiades – groups of interrelated morphological traits, which detects stable relationships among characters. The method helps distinguish conservative taxonomic traits from those dependent on environmental conditions

It should be noted that I. scariosa is characterized by high variability of morphological traits, especially, like the closely related I. pumila, in flower coloration. The species was described from the westernmost margin of its main range based on a specimen collected by Pallas east of the Volga River. It occurs predominantly in the northwestern part of the Caspian Lowland and in the Eastern Ciscaucasia; it is an endemic Caspian European–Caucasian species (Red Book…, 2024). The taxonomic position of the closely related species I. scariosa and I. glaucescens Bunge presents certain difficulties. Both species are either synonymized or considered separate (Tsvelev, 1979; Alexeeva, 2020), with I. scariosa being the preferred species. I. glaucescens was described in Central Asia. The species is represented by small populations found in Russia at the edge of its range in the south of Western Siberia. Outside of Russia, it is known in northeastern Kazakhstan, northwestern Mongolia, and China (Alexeeva, 2020). A similar trend is observed in the Southern Urals: in the Republic of Bashkortostan, I. scariosa is included in the regional Red Book (Red Book…, 2021–2024); in the neighboring Chelyabinsk Region, I. glaucescens is also listed (Kulikov, 2005). Thus, the taxonomic distinction between these closely related species is challenging. There is evidence in the literature, supported by molecular, morphological, and palynomorphological analysis, that I. glaucescens, as well as I. timofejewii and I. curvifolia, are synonyms of I. scariosa (Boltenkov, Artyukova, 2024).

As follows from the dendrogram of species similarities-differences based on the identified diagnostic markers, the closest phylogenetic relationships are observed between the following pairs of species: I. carthaliniae and I. spuria, I. sibirica and I. sanguinea, I. pumila and I. scariosa. Their affinity is also evident in the general habit of the plants. In this way, the pair I. carthaliniae and I. spuria consists of tall plants characterized by long rhizomes with thickened segments, robust, branched stems, broad sword-shaped leaves, and multi-flowered inflorescences (3–5 flowers). The pair I. sibirica and I. sanguinea comprises medium-sized irises with short rhizomes bearing narrow segments, hollow, weakly branched stems, narrow linear leaves arranged in basal clumps, and few-flowered inflorescences (2–3 flowers). The pair I. pumila and I. scariosa includes low-growing plants with thick creeping rhizomes, bearing very short stems, broad-linear or lanceolate leaves in a basal tuft, and large (relative to plant size), few (1–2) flowers positioned close to the ground.

**Molecular phylogenetic analysis of species from the genus Iris**

The results of morphological analysis showed that I. sibirica and I. sanguinea are very close to each other, and this fact was confirmed by the results of molecular phylogenetic analysis. Moreover, according to the obtained data, we can assume that I. sibirica and I. sanguinea are the same species, since the trnL-trnF sequences of all analyzed samples of these species are identical, regardless of the collection location. Other researchers had previously come to this conclusion by analyzing a number of morphological characteristics and using molecular phylogenetic data (Boltenkov et al., 2020).

A very similar situation is observed in the second pair of closely related species – I. pumila and I. scariosa. All analyzed I. pumila specimens, both those with yellow and those with purple flowers, have identical trnL-trnF sequences. The species is polychrome and is characterized by color polymorphism, which is determined by the normal reaction of individuals to environmental conditions (Kashin et al., 2022), and variations in the color of the perianth segments are not associated with molecular genetic polymorphism of populations.

Three sequence variants have been identified in I. scariosa. The first variant (from the Republic of Dagestan) is completely identical to the trnL-trnF sequence of I. pumila, which likely indicates that it is I. pumila. The other two variants differ from I. pumila by substitutions of several nucleotides. The second variant of I. scariosa was found in samples from the Republic of Kazakhstan and the Altai Republic, and the third variant was found in samples from the Republic of Bashkortostan and the Orenburg Region, and the last two variants differ from each other by a single nucleotide substitution. Thus, a comparative analysis of the noncoding sequences of the trnL-trnF region of the chloroplast DNA of I. scariosa from natural populations, as well as an analysis of literary data, shows that this species is correctly identified in the Republic of Bashkortostan and the Orenburg Region.

The situation is completely different in the third pair of related species – I. setosa and I. pseudacorus. First of all, these are evidently two different, albeit closely related, species, which is clearly visible on the phylogenetic tree and has been previously noted by other authors (Choi, Lee, 2024). However, these species differ from each other in terms of intraspecific variability. All I. pseudacorus samples analyzed showed identical trnL-trnF sequences, while five I. setosa samples analyzed showed the presence of two types of trnL-trnF sequences, with all samples collected either in China or Korea. I. setosa is heterogeneous and taxonomically quite clearly delimited from other species, because of this new species and intraspecific taxa have been identified from its composition (Ilyushko et al., 2001). N.B. Alexeeva (2013b) came to the conclusion that the polymorphic complex of I. setosa consists of five species that differ in morphology and ecology. Of course, to verify this, more factual material needs to be analyzed.

All five trnL-trnF sequences of I. lactea are identical to each other, regardless of their habitat. Iris Linnaeus ser. Lacteae Doronkin (Doronkin, 1990) includes species distributed in the temperate Asian regions of the Northern Hemisphere (Rodionenko, 2006; Boltenkov, 2018). Initially, I. lactea was classified in the subgenus Limniris, section Limniris (Rodionenko, 1961; Mathew, 1989), then in the subgenus Eremiris, section Haloiris (Doronkin, 2006). Subsequently, it began to be considered as part of a separate genus Eremiris (Rodionenko, 2006), with which C.A. Wilson (2011), based on molecular genetic studies, does not agree. For a long time, it was believed that the Lacteae Doronkin series was represented by only one polymorphic species, but molecular studies have confirmed phylogenetic branching in at least three lineages corresponding to the taxa I. lactea, I. oxypetala and I. tibetica (Boltenkov et al., 2018b).

Thus, we can state that morphological and molecular phylogenetic analyses show the same results regarding the phylogeny analysis of the studied species of the genus Iris, which means that the identified morphological indicators work in a taxonomic direction.

## Conclusion

Based on the conducted assessment of the structure of morphological variability of representatives of the genus Iris, 10 taxonomic indicators were identified: the length and width of the outer perianth lobes, the length and width of the inner perianth lobes, the length of the filament, anther and pistil, the width of the fruit, and the length and width of the seed.

The establishment of phylogenetic relationships of the studied species based on the identified taxonomic indicators and the molecular phylogenetic analysis based on trnL-trnF, matK and trnS-trnG chloroplast DNA made it possible to combine the studied Iris species into several phylogenetic groups. Thus, it can be concluded that both morphological and molecular phylogenetic analyses yield consistent results regarding the phylogeny of the studied species of the genus Iris, which indicates that the identified morphological indicators reliably demonstrate taxonomic validity.

## Conflict of interest

The authors declare no conflict of interest.
